# Calcium Release-Activated Calcium (CRAC) Channel Inhibition Suppresses Pancreatic Ductal Adenocarcinoma Cell Proliferation and Patient-Derived Tumor Growth

**DOI:** 10.3390/cancers12030750

**Published:** 2020-03-22

**Authors:** Husain Yar Khan, Gabriel B. Mpilla, Rachel Sexton, Srikant Viswanadha, Kumar V. Penmetsa, Amro Aboukameel, Maria Diab, Mandana Kamgar, Mohammed Najeeb Al-Hallak, Mark Szlaczky, Anteneh Tesfaye, Steve Kim, Philip A. Philip, Ramzi M. Mohammad, Asfar S. Azmi

**Affiliations:** 1 Department of Oncology, Wayne State University School of Medicine, Detroit, MI 48201, USA; khanh@karmanos.org (H.Y.K.); mpillag@karmanos.org (G.B.M.); sextonr@karmanos.org (R.S.); kameelo@karmanos.org (A.A.); diabm@karmanos.org (M.D.); kamgarm@karmanos.org (M.K.); hallakm@karmanos.org (M.N.A.-H.); szlaczky@karmanos.org (M.S.); tesfayea@karmanos.org (A.T.); kims@karmanos.org (S.K.); philipp@karmanos.org (P.A.P.); mohammad@karmanos.org (R.M.M.); 2 Rhizen Pharmaceuticals SA, 2300 La Chaux-de-Fonds, Switzerland; srikantv@incozen.com (S.V.); kvp@rhizen.com (K.V.P.)

**Keywords:** CRAC channel, Orai1, STIM, pancreatic cancer, novel therapy

## Abstract

Pancreatic ductal adenocarcinoma (PDAC) remains an unmet clinical problem in urgent need of newer molecularly driven treatment modalities. Calcium signals, particularly those associated with calcium release-activated calcium (CRAC) channels, are known to influence the development, growth, and metastasis of many cancers. This is the first study investigating the impact of CRAC channel inhibition on PDAC cell lines and patient-derived tumor models. PDAC cell lines were exposed to a novel CRAC channel inhibitor, RP4010, in the presence or absence of standard of care drugs such as gemcitabine and nab-paclitaxel. The in vivo efficacy of RP4010 was evaluated in a hyaluronan-positive PDAC patient-derived xenograft (PDx) in the presence or absence of chemotherapeutic agents. Treatment of PDAC cell lines with single-agent RP4010 decreased cell growth, while the combination with gemcitabine/nab-paclitaxel exhibited synergy at certain dose combinations. Molecular analysis showed that RP4010 modulated the levels of markers associated with CRAC channel signaling pathways. Further, the combination treatment was observed to accentuate the effect of RP4010 on molecular markers of CRAC signaling. Anti-tumor activity of RP4010 was enhanced in the presence of gemcitabine/nab-paclitaxel in a PDAC PDx model. Our study indicates that targeting CRAC channel could be a viable therapeutic option in PDAC that warrants further clinical evaluation.

## 1. Introduction

Pancreatic ductal adenocarcinoma (PDAC) is the fourth major cause of cancer-associated mortality in the United States of America (USA). The current 5-year survival rate for PDAC stands at a meager 9% [1]. PDAC is a lethal disease with currently no reliable screening methods to detect it in premalignant or early stages. Moreover, owing to the asymptomatic nature, PDAC is usually diagnosed at a late stage, when the disease has metastasized. This further undermines the success of any treatment. Since surgical resection of PDAC is not feasible in the majority of the cases, radiotherapy and chemotherapy remain the only viable treatment options. However, in the majority of cases, patients develop resistance, which makes identifying novel cellular and molecular targets and better drugs to treat PDAC even more important.

Calcium (Ca^2+^) signaling has been proposed to play a significant role in the growth, invasion, angiogenesis, and metastasis as well as drug resistance of PDAC [2]. An important mechanism of Ca^2+^ influx in non-excitable cells is the store-operated Ca^2+^ entry (SOCE), which gets activated as a result of the depletion of intracellular Ca^2+^ stores in the endoplasmic reticulum (ER) that triggers the opening of Ca^2+^ channels in the plasma membrane. Ca^2+^ release-activated Ca^2+^ (CRAC) channel is the best characterized prototypical SOCE channel. It was first described in T cells and mast cells [3,4] and has since been reported in many different cell types. CRAC channels are composed of protein multimers called Orai, predominantly Orai1. Orai1 is a tetra-spanning plasma membrane protein which is activated by stromal interaction molecules (STIM), located on the ER membrane. STIMs act as sensors of intracellular Ca^2+^ store depletion from the ER. They respond by oligomerizing and translocating to the junction of the ER and plasma membrane where they conjugate with the cytoplasmic domain of Orai proteins, thereby opening the CRAC channel and allowing the influx of extracellular Ca^2+^ into the cell [5].

Several studies have demonstrated the critical role of the CRAC channel components, Orai1 and STIM1, in the proliferation, apoptosis resistance, invasion, and metastasis of various cancer types, including cervical cancer [6], breast cancer [7,8], prostate cancer [9], glioblastoma [10,11], esophageal cell carcinoma [12], hepatocellular carcinoma [13], as well as PDAC [14]. Moreover, STIM1 and Orai1 have been reported to be overexpressed in cancer cells compared to their non-cancerous counterparts [6,12,14]. Therefore, blocking the CRAC channel and its associated signaling pathways may hold promise for the therapy of hard-to-treat cancers, such as PDAC. In fact, the first evidence for the inhibitory effects of calcium channel blockers, verapamil and phenytoin, on the growth of pancreatic cancer cells was provided by Thompson and coworkers in 1994 [15]. However, the possibility of using specific blockers of CRAC channel in PDAC therapy has not been tested thus far.

In this study, we demonstrate that targeting the CRAC channel by the small molecule CRAC channel inhibitor, RP4010, suppresses PDAC proliferation. More significantly, the addition of RP4010 suppressed PDAC patient-derived tumor xenograft (PDx) more effectively when combined with the commonly used PDAC regimen, gemcitabine and nab-paclitaxel. RP4010 is currently being tested in Phase I/Ib clinical trials (ClinicalTrials.gov Identifier: NCT03119467).

## 2. Results

### 2.1. RP4010 Inhibited the Proliferation of Pancreatic Cancer Cells and Suppressed Pancreatic Cancer Cell Colony Formation

**Figure 1:**
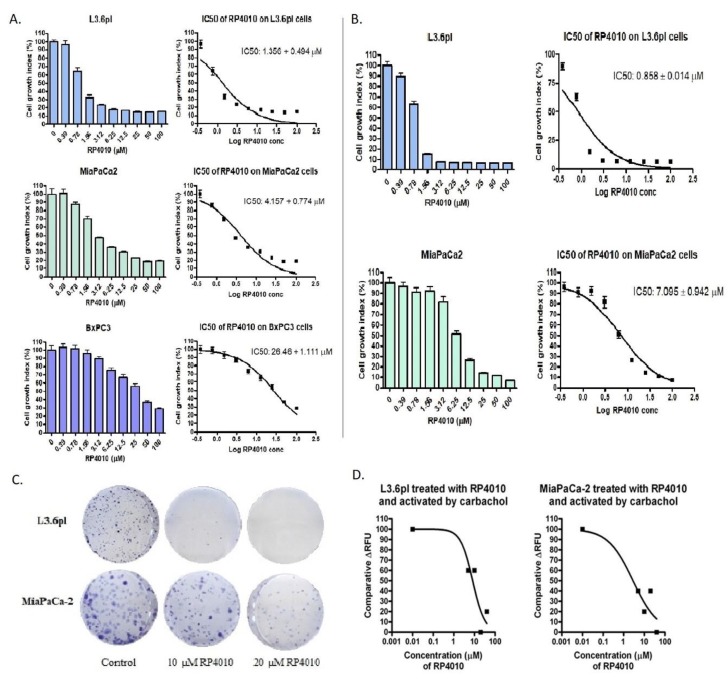
RP4010 suppressed the proliferation of pancreatic cancer cells and inhibited carbachol-induced calcium influx in pancreatic cancer cells. L3.6pl, MiaPaCa-2, and BxPC-3 cells were treated with the indicated concentrations of RP4010 for 72 h. Cell proliferation inhibition was evaluated using MTT assay (**A**) and BrdU assay (**B**), as described in Methods, and average values from three independent experiments were plotted (*n* = 3). Images showing Coomassie Blue stained colonies formed by L3.6pl and MiaPaCa-2 cells (**C**) after treatment with RP4010 at the indicated concentrations (*n* = 3). (**D**) Calcium influx assay was performed as described in Methods, and the relative fluorescence units were plotted for L3.6pl and MiaPaCa-2 cells treated with various concentrations of RP4010 (*n* = 1).

The CRAC channel inhibitor, RP4010, significantly inhibited the proliferation of pancreatic cancer cells, L3.6pl, BxPC-3, and MiaPaCa-2 in a concentration dependent manner. The IC_50_ values of RP4010 for the three pancreatic cell lines ranged from around 1 µM for L3.6pl to approximately 26 µM for BxPC-3 (Figure 1A). BrdU cell proliferation assay further validated the inhibitory effect of RP4010 on L3.6pl and MiaPaCa-2 cells (Figure 1B). In addition, to make sure that the observed suppression of cell proliferation was really due to the inhibition of CRAC channel by RP4010, we performed a siRNA knock down of CRAC channel protein ORAI1 in MiaPaCa-2 cells and tested the effect of RP4010 on the proliferation of such cells by a BrdU assay. As shown in Figure S1, the IC_50_ of RP4010 for ORAI1 knocked down cells (siORAI1) increased by almost 76% as compared with the control cells (32.89 µM vs. 18.7 µM), indicating that the effect of RP4010 on the proliferation of pancreatic cancer cells mainly stems from its ability to inhibit CRAC channel. An RT-qPCR analysis of ORAI1 mRNA expression confirmed the transfection of ORAI1 siRNA in MiaPaCa-2 cells (Figure S2). Furthermore, we found that RP4010 also significantly suppressed the colony formation potential of L3.6pl and MiaPaCa-2 cells (Figure 1C), consistent with the inhibition of cell proliferation by RP4010.

### 2.2. RP4010 Inhibited Carbachol-Induced Calcium Influx in Pancreatic Cancer Cells

Calcium influx assays were conducted to evaluate the mechanism of action. RP4010 significantly inhibited the calcium influx induced by carbachol in pancreatic cancer cells, L3.6pl and MiaPaCa-2 (Figure 1D). Thus, it appears that the inhibition of cell proliferation and colony formation by RP4010 is mediated through the regulation of CRAC channel.

### 2.3. RP4010 Inhibited Calcium-Regulated Akt/mTOR and NFAT Signaling

**Figure 2:**
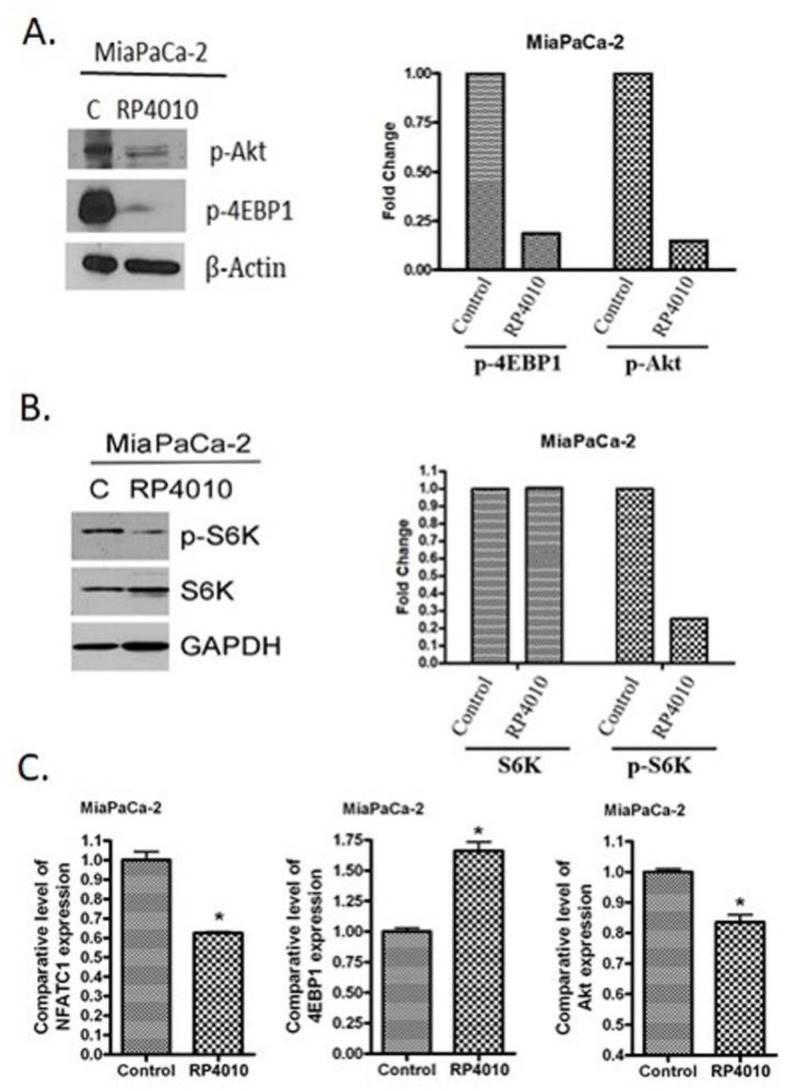
RP4010 inhibited calcium-regulated Akt/mTOR and NFAT signaling. (**A** and **B**) MiaPaCa-2 cells were grown overnight in 100-mm petri dishes to nearly 50% confluence. The cells were then treated on the following day with RP4010 (10 µM) for 72 h. Protein extraction, determination of protein concentration, SDS-PAGE, and Western blot were performed as described in the Methods (*n* = 1). β-actin and GAPDH were used as loading controls. The expression of marker proteins was indicated as fold change relative to the control, and the quantitative analysis of mean pixel density of the blots was performed using ImageJ software. (**C**) MiaPaCa-2 cells were exposed to RP4010 (10 µM) for 48 h. At the end of the treatment period, RNA was isolated and RT-qPCR was performed as described in Methods (* *p* < 0.05).

**Figure 3:**
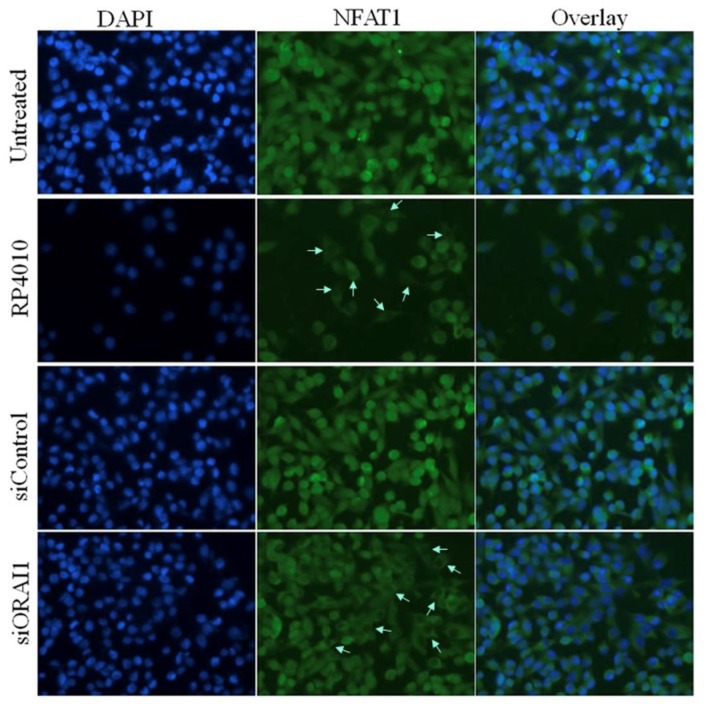
RP4010 impairs the nuclear translocation of NFAT1. MiaPaCa-2 cells were grown on chambered slides and treated with RP4010 (50 µM) for 24 h. Also, MiaPaCa-2 cells having ORAI knock down (siORAI) or its respective control (siControl) were similarly cultured, but received no treatment. Immunofluorescence analysis for NFAT1 nuclear translocation was performed as described in Methods and images were captured at 40× magnification. Arrows in the images indicate the impairment of the relocation of NFAT in the nucleus.

Since CRAC signaling regulates the molecular signal transduction in several important pathways including Akt/mTOR, NFAT, and NF-κB [16,17], we examined the effects of RP4010 on the expression of markers in these pathways at RNA and protein levels. We found that RP4010 induced a reduction in the expression of phosphorylated Akt and 4EBP1 proteins (Figure 2A). RP4010 also reduced the expression of phosphorylated S6K, which is an important molecule in Akt/mTOR signaling (Figure 2B). Figure 2C shows that RP4010 decreased the levels of NFATC1 and Akt mRNAs and increased the level of 4EBP1 mRNA in pancreatic cancer cells. Furthermore, we have demonstrated that RP4010 could impair the translocation of NFAT1 to the nucleus (Figure 3), suggesting that inhibition of CRAC channel by RP4010 can impede calcium signaling, which plays a critical role in the nuclear translocation of NFAT. In order to ensure that this effect of RP4010 is due to its ability to inhibit CRAC channel, we knocked down CRAC channel protein ORAI1 expression in MiaPaCa-2 cells through siRNA. Interestingly, a similar reduction in NFAT1 nuclear translocation was observed in such ORAI1 silenced (siORAI1) cells (Figure 3), confirming that blocking CRAC channel can indeed cause decline in calcium signaling and associated NFAT nuclear translocation. Thus, it is inferred from these results that RP4010 inhibits cancer cell proliferation and colony formation through the inhibition of calcium-regulated Akt/mTOR and NFAT signaling.

### 2.4. RP4010 and Gemcitabine/Nab-Paclitaxel Showed Synergistic Effects on the Inhibition of Cell Proliferation In Vitro with Downregulation of mTOR and NFAT/NF-κB Signaling

**Figure 4:**
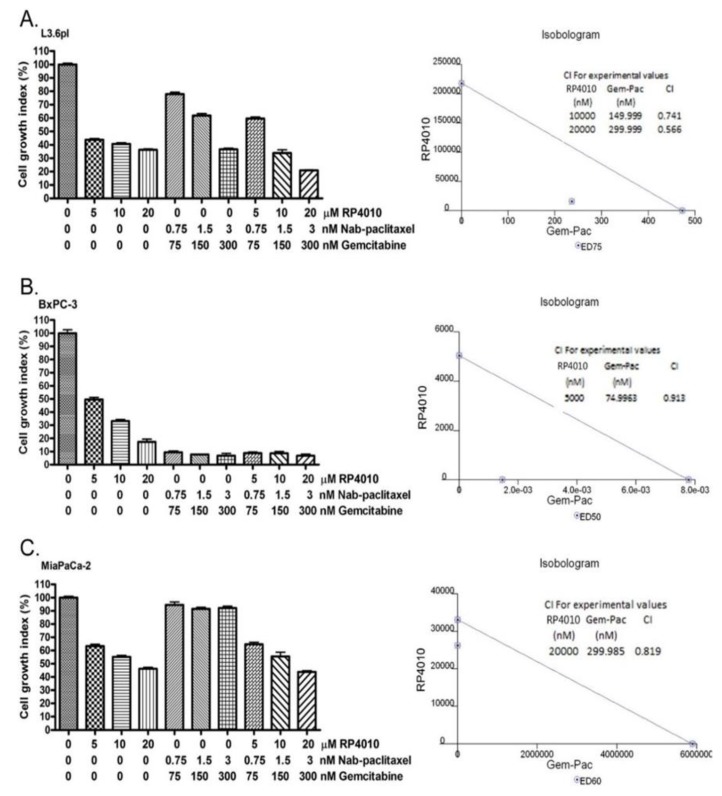
RP4010 and gemcitabine/nab-paclitaxel showed synergistic effects on the inhibition of cell proliferation in vitro. L3.6pl (**A**), BxPC-3 (**B**) and MiaPaCa-2 (**C**) cells were exposed to the indicated concentrations of either RP4010 alone or gemcitabine/nab-paclitaxel, or a combination of RP4010 with gemcitabine/nab-paclitaxel for 72 h, and cell proliferation was evaluated by MTT assay as described in Methods (*n* = 2). CalcuSyn software was employed to generate isobolograms (shown on the right-hand side panel) and determine CI values from the resulting data.

Since RP4010 inhibited cancer cell proliferation and colony formation through the inhibition of calcium-regulated Akt/mTOR and NFAT/NF-κB signaling, we further tested whether RP4010 could enhance the anticancer activities of gemcitabine and nab-paclitaxel, which are commonly used for the treatment of pancreatic cancer. Indeed, we found that a combination of RP4010 with gemcitabine and nab-paclitaxel resulted in enhanced anticancer activities in L3.6pl, BxPC-3, and MiaPaCa-2 (Figure 4A–C) cells. Isobologram analysis showed that the combination of RP4010 and gemcitabine/nab-paclitaxel exerted synergistic effects on the inhibition of cancer cell proliferation with a combination index (CI) less than 1 indicating synergy at the given dose combinations. While it is clear that all the three pancreatic cancer cell lines tested in this experiment showed growth inhibition upon treatment with RP4010, each cell line exhibited a varying degree of sensitivity to gemcitabine/nab-paclitaxel. This is understandable as cell lines differ in their responsiveness to certain treatments owing to possible variations in their molecular and genetic signatures. It may also be mentioned that the synergistic effect on cell growth inhibition (as marked by a CI value < 1 from the isobolograms for the respective cell lines) was not observed at all the dose combinations tested. Specifically, such a synergistic effect was visible in both L3.6pl and MiaPaCa-2 cells when 20 µM RP410 was used in combination with 300 nM gemcitabine and 3 nM nab-paclitaxel. Nevertheless, the observation that RP4010 exhibited synergy (CI < 1) at certain dose combinations with gemcitabine/nab-paclitaxel encouraged us to explore this combination further.

**Figure 5:**
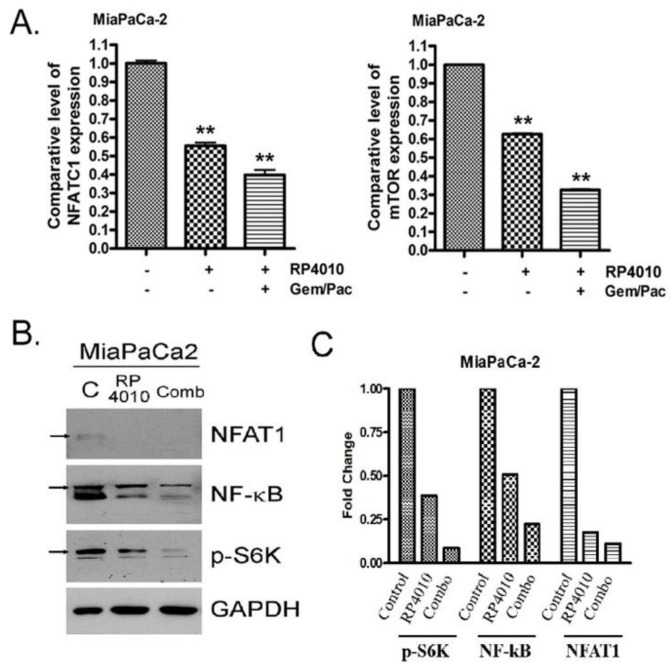
The synergistic effects on the inhibition of cell proliferation by RP4010 and gemcitabine/nab-paclitaxel are mediated through the downregulation of mTOR and NFAT/NF-κB signaling. (**A**) MiaPaCa-2 cells were treated with either 10 µM RP4010 alone or in combination with 300 nM gemcitabine and 3 nM nab-paclitaxel. After 48 h, RNA was isolated and RT-qPCR was performed as described in Methods (** *p* < 0.01). (**B**) MiaPaCa-2 cells were grown overnight in 100-mm petri dishes to around 50% confluence. On the next day, the cells were then incubated with 10 µM RP4010 for 72 h. Extraction of total protein and determination of its concentration were carried out as described in the Methods. The protein lysates were subjected to Western blotting using antibodies against NFAT1, NF-kB, and p-S6K. GAPDH was used as a loading control (*n* = 1). (**C**) Expression of each protein was indicated as fold change relative to the control and a quantitative analysis of mean pixel density of the blots was performed using ImageJ software.

Furthermore, molecular analysis showed that RP4010 combined with gemcitabine and nab-paclitaxel significantly inhibited the expression of NFATC1 and mTOR mRNA (Figure 5A). RP4010 also downregulated the expression of NFAT1, NF-κB, and p-S6K proteins (Figure 5B,C). Such downregulation was more prominent when MiaPaCa-2 cells were treated with RP4010 in combination with gemcitabine/nab-paclitaxel. This indicates that the synergistic effects exerted by RP4010 and gemcitabine/nab-paclitaxel on the inhibition of cell proliferation are mediated via the downregulation of mTOR, NFAT, and NF-κB signaling.

### 2.5. Anticancer Activity of RP4010 Is Potentiated by Gemcitabine/Nab-Paclitaxel in Patient-Derived Xenograft

**Figure 6:**
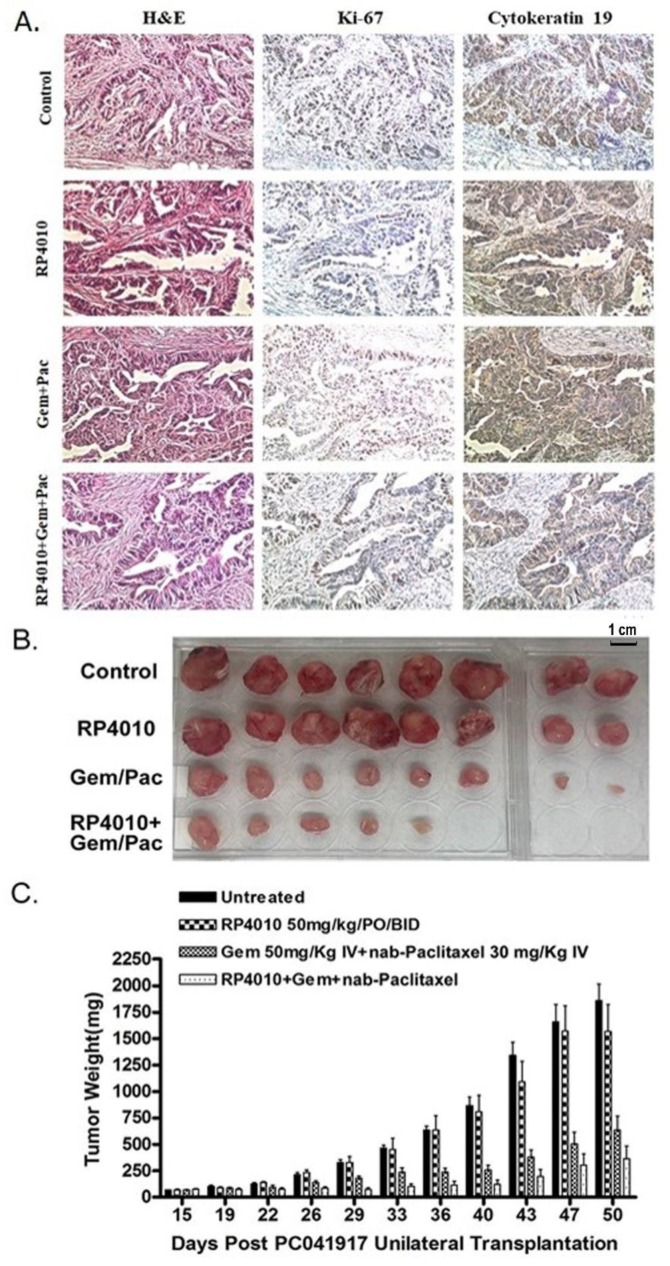
RP4010 potentiated the anti-cancer activity of gemcitabine/nab-paclitaxel in patient-derived xenograft (PDx). (**A**) H&E and immunostaining of tissue sections of the PDx tumors harvested from mice who received different treatments as well as from untreated controls. Images were taken at 200× magnification. (**B** and **C**) The mice with PDx were treated with either RP4010 or gemcitabine/nab-paclitaxel, or a combination of RP4010 with gemcitabine/nab-paclitaxel, and the effect of these treatments on tumor size and weight was assessed up to 50 days post-transplantation of PDx. Treatment started at 15 days post-transplantation when the tumors were palpable. All the tumors were collected 50 days post-transplantation at the culmination of the experiment.

Translation of in vitro activity into in vivo efficacy was studied in a pancreatic cancer patient-derived xenograft (PDx) model. The PDx grown in SCID mice showed strong staining of human cytokeratin 19 and Ki-67, demonstrating the growth of the tumor. However, a decrease in the immunostaining of Ki-67 was observed in the PDx tumors harvested from mice which received a treatment of RP4010 as a single agent or in combination with gemcitabine/nab-paclitaxel (Figure 6A). Importantly, we found that the anti-tumor growth ability of RP4010 was potentiated when it was used in combination with gemcitabine/nab-paclitaxel in PDx (Figure 6B,C). It may be noted that up to 40 days post-transplantation of PDx, RP4010 did not exhibit any apparent change in tumor weight. Nevertheless, some noticeable regression in tumor weight was observed after 40 days post-transplantation. This indicates that the effect of RP4010 as a single agent started to appear late while its combination treatment could show conspicuously potent effect in reducing tumor weight from relatively early on after the start of treatment (Figure 6C). An unpaired two tailed Student’s t-test confirmed that the tumor growth inhibition induced by the triple combination treatment was statistically significant (*p* = 0.0097; Confidence Interval (C.I.) = 90%) compared to that by single agent RP4010. Although the double combination is not statistically significant from the triple combination, it is worthwhile to draw attention toward the fact that 3 out of the 8 mice (37.5%) in the triple combination treatment group showed a complete response and were found tumor free (Figure 6B), which suggests that the combination of RP4010 with gemcitabine and nab-paclitaxel is definitely more effective than a combination of gemcitabine and nab-paclitaxel. Therefore, these results confirm the findings from in vitro experiments and suggest that the CRAC channel inhibitor RP4010 could enhance the anti-tumor effects of gemcitabine and nab-paclitaxel.

## 3. Discussion

In this paper we report, for the first time, that targeting the CRAC channel by small molecule drug RP4010 suppresses the growth of PDAC cell lines, and patient-derived tumors. These studies bring forward a novel therapy for the difficult-to-treat PDAC.

CRAC channels are one of the major routes of Ca^2+^ entry in non-excitable cells. A decline in the levels of Ca^2+^, stored in the ER, signals the opening of these channels and entry of extracellular Ca^2+^ into the cell. This influx of Ca^2+^ through CRAC channels is believed to be involved in the regulation of various cellular and physiological processes [18], including the ones associated with cancer development, such as proliferation and apoptosis of cancer cells, metastasis and tumor neovascularization [19]. The ability to mediate multiple events related with cancer makes CRAC channel a promising target for anticancer therapy. Aberrant activity and expression of CRAC channel has been reported not just in cancer, but also in a number of other disorders [20]. Thus, an increased realization that the inhibition of CRAC channel may have significant clinical implications has ignited a considerable interest in developing small molecule inhibitors of this channel.

SKF-96365 was the first CRAC channel inhibitor which exhibited in vitro and in vivo anticancer effects in breast [7], cervical [6], and esophageal cancer [12] models. SKF-96365 was, however, found to be non-selective for CRAC channel and could inhibit other Ca^2+^ channels as well [21]. Although a number of other CRAC channel inhibitors have been developed over the years, a majority of them unfortunately did not manage to reach clinical trials, mainly due to their high toxicity and poor selectivity [20]. Nevertheless, there are some selective inhibitors of CRAC channel which hold promise for further drug development. There have only been four such inhibitors that have succeeded to enter clinical trials [18]. Among them, only RP4010 continues to remain in clinical development. It is currently in a Phase I/Ib study for evaluation of safety and efficacy in patients with relapsed or refractory non-Hodgkin’s lymphoma (https://clinicaltrials.gov/ct2/show/NCT03119467).

Interestingly, it has been shown that CRAC channel plays a pro-survival and anti-apoptotic role in PDAC cells [14], and its inhibition could plausibly make PDAC cells more susceptible to cell death. CRAC channel inhibition-induced anti-proliferative and anti-metastatic effects have been reported in breast cancer, prostate cancer, and leukemia [10]. In a recent study, Cui et al. have shown that the CRAC channel inhibitor RP4010 could effectively reduce the proliferation of esophagus squamous cell carcinoma cells. It was able to inhibit cell proliferation in a few other types of cancer cells as well, including the doxorubicin resistant ovarian cancer cells [22]. In line with these observations, our study demonstrates that the exposure of PDAC cell lines to RP4010 suppressed proliferation.

RP4010 was found to synergize with gemcitabine and nab-paclitaxel in PDAC cellular models (CI < 1). Our results suggest that the synergistic effect exerted by the combination of RP4010 with gemcitabine and nab-paclitaxel on the proliferation of pancreatic cancer cells was mediated through the downregulation of calcium regulated pro-survival signaling pathways, such as Akt/mTOR and NFAT/NF-kB signaling. However, more work needs to be done to fully understand the mechanistic basis for this synergy. It has been reported that the expression levels of CRAC channel proteins, Orai1 and STIM1, were found to be relatively low in the normal pancreatic ductal epithelial cells when compared to several PDAC cell lines, suggesting that cancer cells probably upregulate Orai1 and STIM1 in order to save themselves from undergoing apoptosis [14]. It must also be mentioned here that the levels of intracellular Ca^2+^ have been reported to be elevated in various human and animal cancer cells [23], which may suggest the possibility of an increased SOCE through CRAC channels in neoplastic cells.

Our observation that the combination of RP4010 with gemcitabine/nab-paclitaxel was effective in suppressing the growth of PDAC PDx is encouraging and strengthens the case for the use of this combination in the clinical setting. Evidence in support of the above contention was provided by a study which found that the use of calcium channel blockers improved the overall survival of pancreatic cancer patients receiving gemcitabine-based therapy [24]. However, another recent study has reported an increased risk of pancreatic cancer to be associated with the use of short-acting calcium channel blockers [25]. Several other studies have shown different calcium channel blockers to suppress cell proliferation and reduce tumor growth in pancreatic cancer models [15,26,27]. It should be noted that the calcium channel blockers investigated in the aforementioned studies are not essentially CRAC channel inhibitors, but rather inhibitors of other types of calcium channels/pumps in the cell. Although the available literature makes it clear that dysregulation of calcium signaling plays an important role in pancreatic cancer, information pertaining to the effects of CRAC channel inhibitors on pancreatic cancer cells is limited.

## 4. Materials and Methods

### 4.1. Cell Lines, Reagents and Antibodies

MiaPaCa-2 and BxPC-3 cells were purchased from American Type Culture Collection (ATCC, Manassas, VA, USA). L3.6pl cells were obtained from MD Anderson Cancer Center. All cells were maintained in DMEM (Invitrogen, Carlsbad, CA, USA) supplemented with 10% fetal bovine serum (FBS), 100 U/mL penicillin, and 100 μg/mL streptomycin in a 5% CO_2_ atmosphere at 37 °C. The cell lines have been tested and authenticated in a core facility of the Applied Genomics Technology Center at Wayne State University. The method used for testing was short tandem repeat (STR) profiling using the PowerPlex^®^ 16 System from Promega (Madison, WI. USA). RP4010 (Rhizen Pharmaceuticals S.A., La Chaux-de-Fonds, Switzerland) was dissolved in DMSO to make a 50-mM stock solution; 1-mM stock solutions of gemcitabine (Gemzar, Eli Lily, Indianapolis, IN, USA) and nab-paclitaxel (Abraxane, Celgene, Summit, NJ, USA) were also made in DMSO. Anti-NFAT1 (Cell Signaling, Danvers, MA, USA), anti-p-Akt (Cell Signaling), anti-p-4EBP1 (Cell Signaling), anti-S6K (Cell Signaling), anti-p-S6K (Cell Signaling), anti-NF-κB (MilliporeSigma, Burlington, MA, USA), anti-β-actin (Cell Signaling), and anti-GAPDH (Thermo Fisher Scientific, Waltham, MA, USA) primary antibodies were used for Western Blot analysis.

### 4.2. MTT Assay and Synergy Analysis

Different pancreatic cancer cells, namely L3.6pl, MiaPaCa-2, and BxPC-3, were seeded in 96-well microtiter culture plates at a density of 5 × 10^3^ cells per well. The growth medium was removed after overnight incubation and replaced with fresh medium containing RP4010 at increasing concentrations (0–100 µM) diluted from a 50-mM stock. After 72 h of exposure to the drug, 20 µL of MTT (3-(4,5-dimethylthiazol-2-yl)-2,5-diphenyltetrazolium bromide) solution (5 mg/mL in PBS) was added to each well and further incubated for 2 h. The supernatant was aspirated from the wells upon termination and the MTT formazan formed by metabolically viable cells was dissolved in 100 µL of isopropanol. The plates were rocked gently on a gyratory shaker for 30 min and the absorbance of the samples was measured at 570 nm on a plate reader SynergyHT (BioTek, Winooski, WI, USA). Based on cell proliferation data, IC_50_ values were calculated using the GraphPad Prism 4 software.

For the synergy analysis, L3.6pl, BxPC-3, and MiaPaCa-2 cells were treated with either 5–20 µM RP4010, or 75–300 nM gemcitabine and 0.75–3.0 nM nab-paclitaxel, or a combination of RP4010 with gemcitabine and nab-paclitaxel at the aforementioned doses for 72 h. Cell growth index was determined using MTT assay as described above. The resulting cell growth data was used to construct isobolograms and calculate combination index (CI) values by the CalcuSyn software (Biosoft, Cambridge, UK).

### 4.3. BrdU Cell Proliferation Assay

Cells were seeded in 96-well culture plates at a density of 5 × 10^3^ cells per well. The growth medium was removed after overnight incubation and replaced with fresh medium containing RP4010 at increasing concentrations (0–100 µM) diluted from a 50 mM stock. After 72 h of exposure to the drug, the effect on cell proliferation was assessed using BrdU cell proliferation ELISA kit (Roche, Mannheim, Germany) according to the manufacturer’s protocol. Briefly, 10 µL of BrdU (5′-bromo-2′-deoxyuridine) labeling solution was added to each well (100 µL) and further incubated for 4 h at 37 °C. Then, the labeling medium was removed and the cells were fixed at room temperature for 30 min, following which the fixing solution was removed and the cells were incubated with enzyme bound anti-BrdU antibody conjugate for 90 min. Finally, the wells were washed thoroughly and substrate solution was added. The subsequent color development was photometrically detected at 370 nm on a SynergyHT (BioTek) plate reader. Based on cell proliferation data, IC_50_ values were calculated using the GraphPad Prism 4 software.

### 4.4. Small Interference RNA and Transfection

MiaPaCa-2 cells were seeded at 5 × 10^4^ cells per well in a 6-well plate and were cultured in antibiotic-free growth medium. The cells were then incubated at 37 °C in a CO_2_ incubator overnight. On the next day, the cells were transfected with either ORAI1 or control siRNA using Lipofectamine 3000 reagent transfection kit (Thermo Fisher Scientific), following the manufacturer’s protocol. After 72 h, the cells were harvested, seeded again, and transfected one more time (double transfection). At the culmination of the second siRNA transfection period, the cells were washed with PBS, collected, counted, and seeded again for BrdU cell proliferation assay or immunofluorescence analysis. RNA was isolated from the remaining population of cells and the siRNA knock down efficiency was analyzed using RT-qPCR.

### 4.5. Cell Colony Formation Assay

MiaPaCa-2 and L3.6pl cells were seeded at a density of 50,000 cells per well in six well plates and treated with 10 and 20 µM RP4010 for 72 h. At the end of the treatment period, cells were trypsinized and 400 cells were re-plated in six well plates for an additional ten days. Plates were stained with Coomassie Blue and colonies were photographed.

### 4.6. Calcium Influx Assay

The calcium influx assays were conducted using the Fluo-4 Direct calcium Assay Kit (Invitrogen) following the protocol provided by the manufacturer. Briefly, MiaPaCa-2 and L3.6pl cells were seeded in black 96-well plates. Cells were treated with different concentrations of RP4010, stimulated with 300 nM carbachol (calcium agonist), and incubated with Fluo-4 Direct calcium assay reagent and probenecid in buffer. The relative fluorescent units (RFU) were measured.

### 4.7. RNA Isolation and mRNA Real-Time RT-qPCR

Total RNAs from cell lines treated with RP4010 or combination were extracted and purified by using the RNeasy Mini Kit and RNase-free DNase Set (QIAGEN, Valencia, CA, USA) following the protocol provided by the manufacturer. The mRNA expression of markers downstream to CRAC channel signaling were analyzed by real-time RT-qPCR using High Capacity cDNA Reverse Transcription Kit and SYBR Green Master Mixture from Applied Biosystems (Waltham, MA, USA). Sequences of primers used are listed in Table 1. The qPCR was initiated by 10 min at 95 °C before 40 thermal cycles, each of 15 s at 95 °C and 1 min at 60 °C in a StepOnePlus real-time PCR system (Applied Biosystems). Data were analyzed according to the comparative Ct method and were normalized to actin and/or 18S rRNA expression in each sample.

### 4.8. Preparation of Total Protein Lysates and Western Blot Analysis

For total protein extraction, pancreatic cancer cells were lysed in RIPA buffer and protein concentration was measured using BCA protein assay (PIERCE, Rockford, IL, USA). Western Blot analysis was conducted to measure the alterations in the protein expression of genes. Briefly, the total proteins were subjected to 10% or 14% SDS-PAGE, and electrophoretically transferred to nitrocellulose membrane. The membranes were incubated with specific primary antibodies, and subsequently incubated with secondary antibody conjugated with peroxidase (Bio-rad, Hercules, CA, USA). The signal was detected using the chemiluminescent detection system (PIERCE). Densitometric analysis of the data was performed using the ImageJ software (NIH, Bethesda, MD, USA).

### 4.9. Immunofluorescence Analysis

Cells cultured on chambered glass slides were fixed with 4% paraformaldehyde for 10 min and subsequently permeabilized with 0.05% Triton-X100 for 15 min. The slide chambers were then washed with PBS and 5% bovine serum albumin (BSA) blocking solution was added. After an hour, slide chambers were washed again with PBS, and anti-NFAT1 rabbit primary antibody (1:50 dilution in 3% BSA) was added and left overnight in the cold room. The next day, slide chambers were washed with PBS-T and PBS before goat anti-rabbit secondary antibody Alexa Fluor^®^ 488 conjugate (Thermo Fisher Scientific) was added at 1:500 dilution in 3% BSA. Finally, ProLong™ Gold antifade reagent with DAPI (Thermo Fisher Scientific) was applied (one drop over each chamber section), coverslip was placed over the slide, and images were captured under an inverted fluorescent microscope EVOS (Thermo Fisher Scientific).

### 4.10. Animal Studies

The PDx mouse model used in this study was generated by transplanting human primary pancreatic ductal adenocarcinoma tumor unilaterally into the flank of 35 ICR-SCID female mice (Taconic Biosciences). The tumor was obtained from a 74-year old male patient who underwent Whipple’s procedure at Karmanos Cancer Center which showed pT2 pN1 M0. The patient received no chemotherapy prior to surgery. Once tumor uptake was confirmed (about two weeks post-implantation), mice were randomized and assigned to 4 groups with 8 per cohort; group 1: untreated, group 2: RP4010 orally- treated (50 mg/kg), group 3: gemcitabine and nab-paclitaxel intravenously-treated (50 mg/kg and 30 mg/kg respectively), and group 4: triple combination treated. RP4010 was administered twice daily for three weeks while clinical grade gemcitabine and nab-paclitaxel were injected once a week for the same duration. Immunohistochemistry was performed on formalin fixed tumor sections collected at the end of experiment to confirm the human ductal characteristic. All animal work was performed under Wayne State University approved IACUC protocol (Approval no. 16-03-059) and human IRB approval for PDx (Approval no. 2016-029) was also obtained.

### 4.11. Immunostaining

Paraffin sections of the PDx tumors were processed and stained with H&E and antibodies in a core facility at the Department of Oncology, Wayne State University. The following antibodies were used for immunohistochemistry staining: Ki67 (Cell Marque, Rocklin, CA, USA) and cytokeratin 19 (Abcam, Cambridge, MA, USA).

### 4.12. Statistics

Wherever appropriate, the data were subjected to a Student’s t-test using GraphPad Prism 4 software (La Jolla, CA, USA), and *p* < 0.05 was considered statistically significant.

## 5. Conclusions

In conclusion, this study demonstrated the therapeutic potential of RP4010, a small molecule CRAC channel inhibitor, for inhibiting the proliferation of PDAC cells in culture and reducing the growth of patient-derived PDAC tumor xenografts in mice, both as a single agent as well as in combination with gemcitabine and nab-paclitaxel. The synergy with gemcitabine/nab-paclitaxel was linked to a corresponding change in the expression of various markers of downstream signaling pathways associated with the CRAC channel.

## Figures and Tables

**Table 1:** Sequences of primers used.

Primers	Sequences	
NFATC1	Forward	TGTCTGGGAGATGGAAGCGA
	Reverse	CGGTTAGAAAGATGGCGTTACC
Akt	Forward	TTGTGAAGGAGGGTTGGCTG
	Reverse	CTCACGTTGGTCCACATCCT
mTOR	Forward	TTCCGACCTTCTGCCTTCAC
	Reverse	CCACAGAAAGTAGCCCCAGG
4EBP1	Forward	CAAGGGATCTGCCCACCATT
	Reverse	ACACGATGGCTGGTGCTTTA
ORAI1	Forward	GAGGTGATGAGCCTCAACGAG
	Reverse	TAGTCGTGGTCAGCGTCCAG
actin	Forward	GCACAGAGCCTCGCCTT
	Reverse	TCATCATCCATGGTGAGCTG
18S	Forward	GCAATTATTCCCCATGAACG
	Reverse	GGCCTCACTAAACCATCCAA

## Supplementary Materials

The following are available online at https://www.mdpi.com/2072-6694/12/3/750/s1, Figure S1: Effect of RP4010 on the proliferation of ORAI1 knocked down cells, Figure S2: Transfection of siORAI1 in MiaPaCa-2 cells leads to knock down of ORAI1 mRNA.
