# Supplementary material for: Calcium Release-Activated Calcium (CRAC) Channel Inhibition Suppresses Pancreatic Ductal Adenocarcinoma Cell Proliferation and Patient-Derived Tumor Growth
Source: Cancers (Basel). 2020 Mar 22;12(3):750. doi: 10.3390/cancers12030750 (PMC7140111; doi:10.3390/cancers12030750)
Supplement: Supplementary file 1 [file cancers-12-00750-s001.zip › cancers-626349 supplementary final/cancers-626349 supplementary layout.pdf]

cancers

Supplementary Meterials:

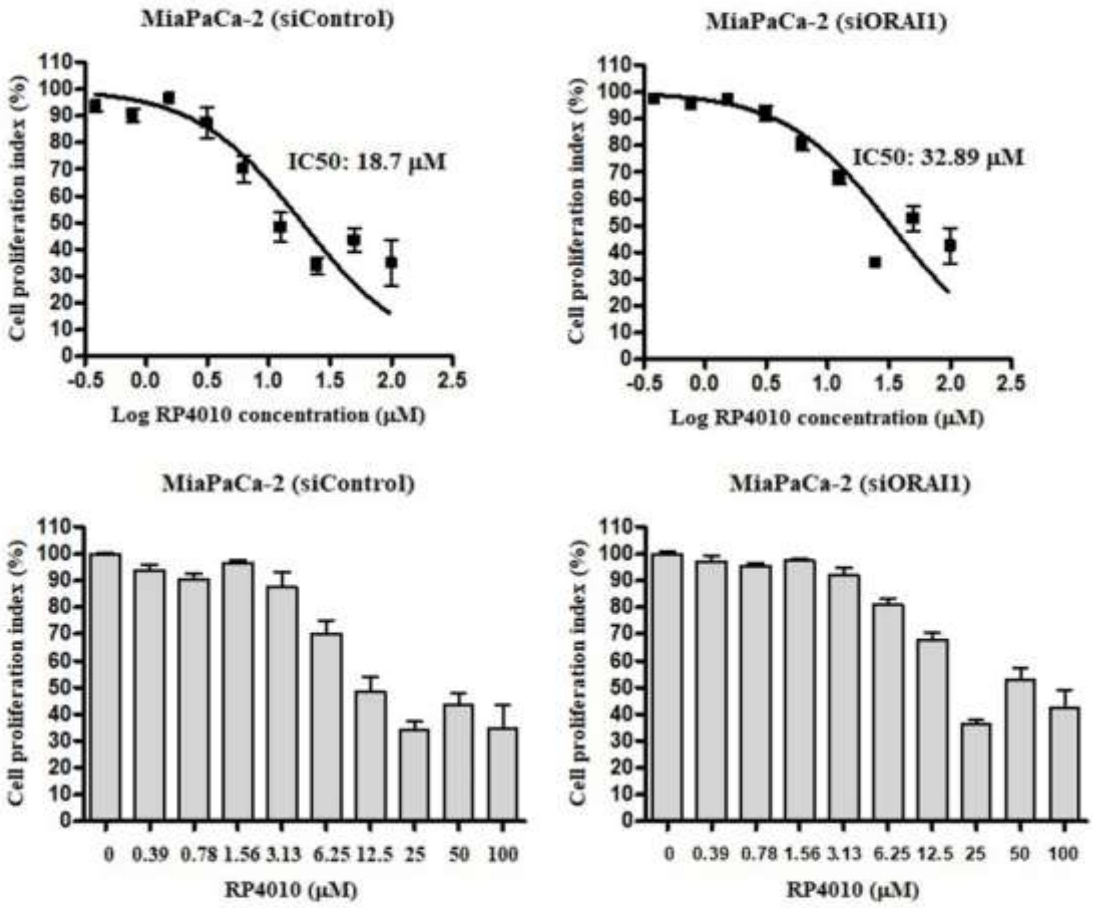

Figure S1. Effect of RP4010 on the proliferation of ORAI1 knocked down cells. Both siControl and siORAI1 transfected MiaPaCa-2 cells were seeded at 5000 cells/well in 96-well plates and incubated overnight at 37 °C. The cells were then exposed to different concentrations of RP4010 for 72 h, following which BrdU assay was performed as described in Methods and IC50 values were obtained using GraphPad Prism 4 software.

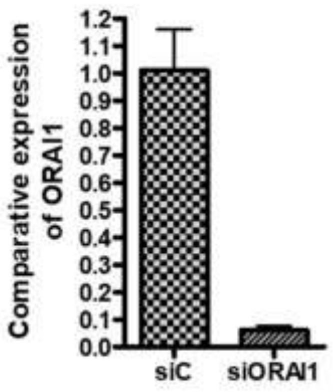

Figure S2. Transfection of siORAI1 in MiaPaCa-2 cells leads to knock down of ORAI1 mRNA. MiaPaCa-2 cells were transfected with either siControl or siORAI1 as described in Methods and their total RNA was isolated to perform RT-qPCR analysis for ORAI1 mRNA expression.
