# Supplementary material for: Calcium Release-Activated Calcium (CRAC) Channel Inhibition Suppresses Pancreatic Ductal Adenocarcinoma Cell Proliferation and Patient-Derived Tumor Growth
Source: Cancers (Basel). 2020 Mar 22;12(3):750. doi: 10.3390/cancers12030750 (PMC7140111; doi:10.3390/cancers12030750)
Supplement: Supplementary file 1 [file cancers-12-00750-s001.zip › cancers-626349 supplementary final/cancers-626349 Western Blot.pptx]

## Slide 1
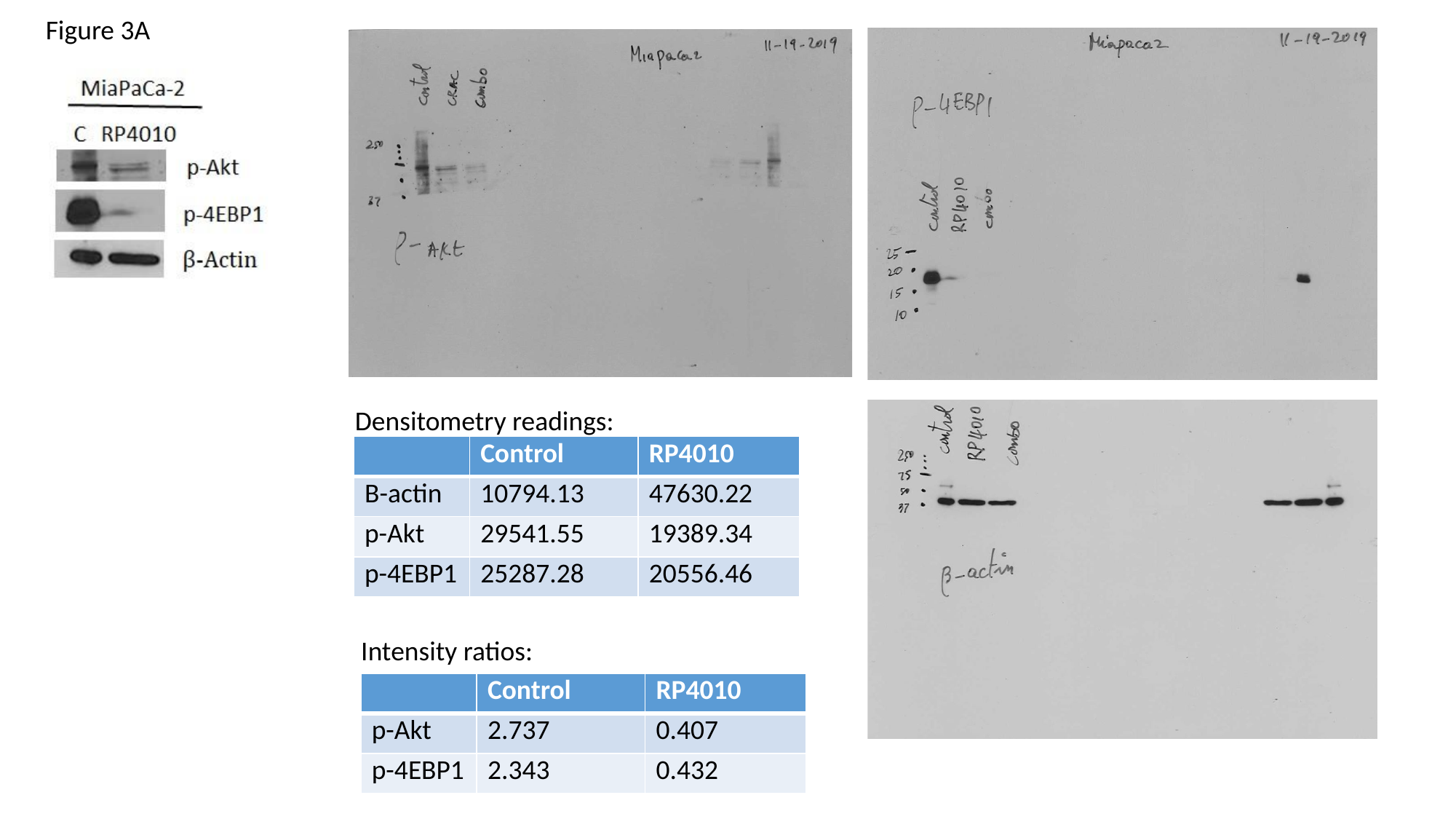

Figure 3A
Densitometry readings:
| | Control | RP4010 |
| --- | --- | --- |
| Β-actin | 10794.13 | 47630.22 |
| p-Akt | 29541.55 | 19389.34 |
| p-4EBP1 | 25287.28 | 20556.46 |
Intensity ratios:
| | Control | RP4010 |
| --- | --- | --- |
| p-Akt | 2.737 | 0.407 |
| p-4EBP1 | 2.343 | 0.432 |

## Slide 2
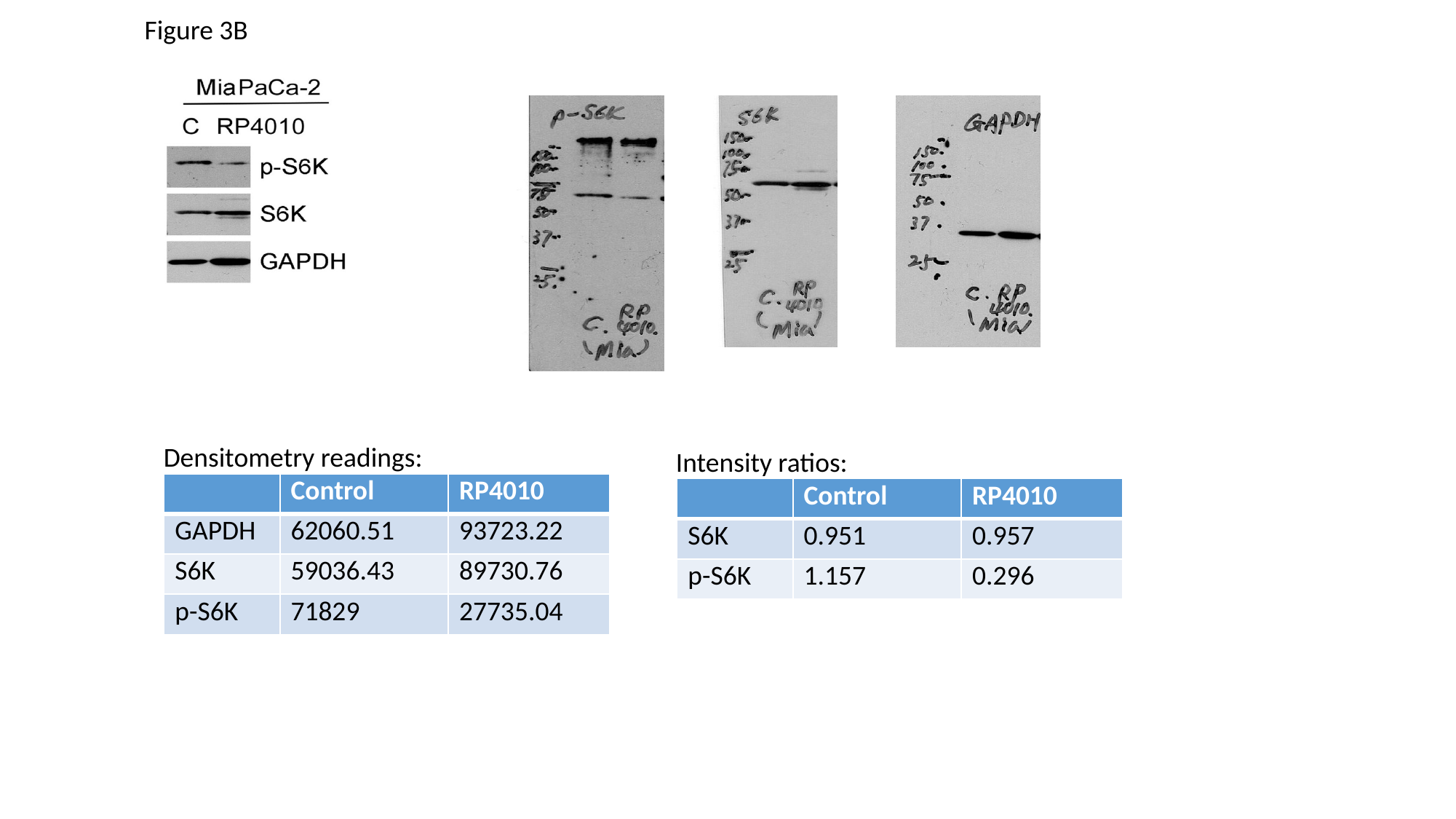

Figure 3B
Densitometry readings:
Intensity ratios:
| | Control | RP4010 |
| --- | --- | --- |
| GAPDH | 62060.51 | 93723.22 |
| S6K | 59036.43 | 89730.76 |
| p-S6K | 71829 | 27735.04 |
| | Control | RP4010 |
| --- | --- | --- |
| S6K | 0.951 | 0.957 |
| p-S6K | 1.157 | 0.296 |

## Slide 3
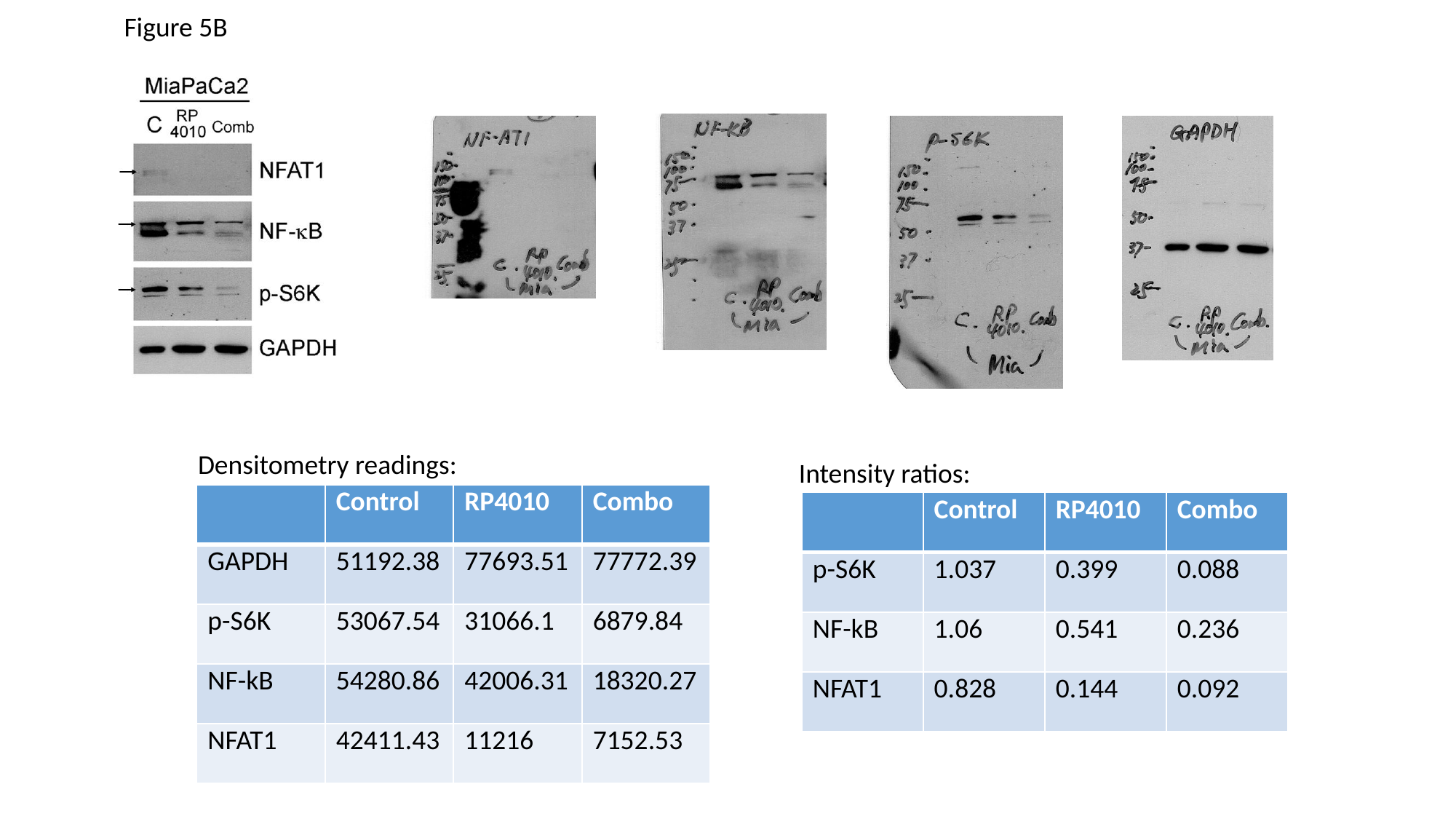

Figure 5B
Densitometry readings:
Intensity ratios:
| | Control | RP4010 | Combo |
| --- | --- | --- | --- |
| GAPDH | 51192.38 | 77693.51 | 77772.39 |
| p-S6K | 53067.54 | 31066.1 | 6879.84 |
| NF-kB | 54280.86 | 42006.31 | 18320.27 |
| NFAT1 | 42411.43 | 11216 | 7152.53 |
| | Control | RP4010 | Combo |
| --- | --- | --- | --- |
| p-S6K | 1.037 | 0.399 | 0.088 |
| NF-kB | 1.06 | 0.541 | 0.236 |
| NFAT1 | 0.828 | 0.144 | 0.092 |
